# Energy Consumption and Carbon Emissions of Compressed Earth Blocks Stabilized with Recycled Cement

**DOI:** 10.3390/ma18174194

**Published:** 2025-09-06

**Authors:** Alessandra Ranesi, Ricardo Cruz, Vitor Sousa, José Alexandre Bogas

**Affiliations:** CERIS, Department of Civil Engineering, Architecture and Environment, Instituto Superior Técnico, University of Lisbon, Av. Rovisco Pais 1, 1049-001 Lisboa, Portugal; ricardojtcruz@tecnico.ulisboa.pt (R.C.); vitor.sousa@tecnico.ulisboa.pt (V.S.); jose.bogas@tecnico.ulisboa.pt (J.A.B.)

**Keywords:** earth construction, stabilization, Portland cement, recycled cement, energy consumption, carbon emissions

## Abstract

Driven by the pursuit of more sustainable materials, earth construction has seen renewed interest in recent years. However, chemical stabilization is often required to ensure adequate water resistance. While recycled cement from concrete waste (RCC) has recently emerged as a more sustainable alternative to ordinary Portland cement (OPC) for soil stabilization, its environmental impact remains unassessed. A hybrid model, built on collected data and direct simulations, was implemented to estimate energy and carbon emissions of compressed earth blocks (CEBs) stabilized with RCC as a partial or total replacement of OPC. Four operational scenarios were assessed in a cradle-to-gate approach, evaluating the environmental impact per CEB unit, and normalizing it to the CEB compressive strength. OPC CEBs showed up to 9 times higher energy consumption (2.46 vs. 0.24 MJ/CEB) and about 35 times higher carbon emissions (0.438 vs. 0.012 kgCO_2_/CEB) than UCEBs. However, replacing OPC with RCC reduced energy consumption by up to 8% and carbon emissions by up to 64%. Although RCC CEBs showed lower mechanical strength, resulting in higher energy consumption when normalized to compressive strength, carbon emissions remained up to 48% lower compared to OPC CEBs. RCC emerged as a more sustainable alternative to OPC for earth stabilization, while also improving the mechanical strength and durability of UCEBs.

## 1. Introduction

The European Environment Agency (EEA) estimates that a 37% reduction in the European Union (EU) total greenhouse gas (GHG) emissions was achieved between 1990 and 2023 [1]. With the mitigation strategies already implemented in Europe, by 2030, a 43% reduction is expected, with the possibility of boosting it to 49% and getting very close to the 55% reduction target set by the European Commission [2]. However, despite being responsible for only 6% of the total global emissions, the EU is listed in the global GHG emissions report of 2024 [3] as one of the main countries (along with China, U.S., India, Russia, and Brazil) responsible for the increase of 1.9% (or 994 Mt CO_2eq_) of GHG emissions between 2022 and 2023.

Globally, the building sector increased its GHG emissions from 1990 to 2023 by 1%. In the EU, the building sector is responsible for over 35% of the total waste generation, accounting for about 50% of all extracted material [4]. It is expected that a greater material efficiency could save up to 80% of the 5–12% total GHG emissions that the construction (and renovation) sector is responsible for [5]. For instance, in Portugal, the building sector accounted for 6.45% of the GHG emissions in 2023 (3.42 Mt CO_2eq_ out of a total of 53 Mt CO_2eq_).

In this context, academia and industry have been exploring the use of natural or more eco-efficient construction materials. One attempt is the modernization of traditional earth constructions, such as rammed earth, adobe, or cob [6]. Earth is a vernacular construction material with low environmental impact due to its low embodied energy [7,8], traditional proximity to the construction site (no transportation) [9,10], little depth of excavation [11] (possibility of using material resulting from other works, namely foundation execution), and its full reusability if not stabilized [12]. Nowadays, it is estimated that 650–700 million people still live in earth dwellings worldwide (8–10% of the world population), reaching 20–25% of the population in low- or medium-HDI (Human Development Index) countries [13].

Earth construction is conventionally cost-efficient [14]. The production costs tend to be low, since natural, abundant, and local raw materials make up most of the resources needed. Moreover, due to the low level of technology required, construction costs are also low. In high-HDI countries, the labor requirements may represent a cost increase during the construction stage, but this is hindered in low- and medium-HDI countries due to the lower labor costs and the traditional participative nature of the construction process, based on voluntary work [15]. The end-of-life cost is also lower, particularly when not stabilized or bio-stabilized, as in some cases, earth can be easily recovered and reused. However, the shortcomings in durability, namely the susceptibility to liquid water and erosion, imply higher maintenance costs and/or lower lifespan. In a whole life cycle cost analysis, the latter affects all the other costs when comparing with alternative solutions. This underpins the growing interest in finding new stabilizers for earth construction observed over the last 10 years [16,17]. Some agro-industrial wastes and by-products (e.g., rice husk ash, sugarcane molasses, fly ash, and blast furnace slag) have been used as alternatives to the less environmentally friendly Portland cement and hydraulic lime [18,19,20,21]. Still, it must be assessed if the greater durability and mechanical performance of stabilized earth compensates for the additional environmental and financial cost.

According to a recent literature review [22], compressed earth blocks (CEBs) are among the most studied earth-based construction techniques (after rammed earth and adobe), with around 400 research papers on this subject published from 1968 to 2023. In this technique, the earth benefits from its mechanical stabilization through high pressing forces. Chemical stabilization of CEBs with Portland cement started in the 1920s [23], but it was only in 1958, with the patent of the first manual press, the CINVA-Ram machine, that this building product spread [23]. Ordinary Portland cement (OPC) is not a green material, mainly due to the combination of its high production temperature and the substantial carbon content of the raw material, which is released during the burning process [24]. A possible strategy to reduce its environmental impact is to recycle it [25,26,27,28,29,30,31,32] from old cement paste waste through a low-temperature thermoactivation process [33,34,35]. It has been shown that recycled cement (RC) can be engineered to perform as well as OPC in cement-based materials [36,37,38]. Concrete, with up to 40% OPC replacement by RC, shows only minor losses in strength, whereas higher replacement levels lead to reductions due to RC's greater water demand [39].

However, producing RC requires the highly challenging preliminary separation of cement paste from the remaining constituents of hardened concrete. Recently, a technically and environmentally efficient separation method has been developed to recover a high-purity cement fraction (>75 wt%) from concrete waste, which can then be thermoactivated at low temperatures to produce recycled cement [40]. The use of decarbonized raw material (hydrated cement paste) along with the lower processing temperature (650 °C) largely compensates for the processing burden associated with the separation of the cement paste from the aggregates [41,42]. This novel method is based on magnetic separation, which requires the crushed concrete waste to be cleaned of dust. Hereby, the original method resorted to water, but the washing and consequent drying of cement waste hindered most of the energy benefits of recycling. The development of an alternative air cleaning method has already solved this problem and will keep going greener as the electricity-specific carbon emissions lower with the increase in the share of renewable energy sources [43]. In fact, due to the lower temperature required for the process, it would be easier to use only electricity as an energy source to obtain recycled thermoactivated cement than to produce OPC. Considering this strategy, a potential reduction of up to 80% in CO_2_ emissions compared to OPC is reported [43]. Through LCA analysis, Real et al. [41] estimated that the global warming potential could be about 70% lower in RC production than in OPC production. In addition, RC promotes the reuse of construction and demolition waste (CDW) and saves excessive consumption of natural resources in clinker manufacture.

The technical feasibility of recycled cement as an alternative low-carbon substitute for OPC in CEB stabilization was analyzed in previous studies. Due to the higher water demand of RC, recycled cement-stabilized earth blocks (RC CEBs) tend to achieve less density than ordinary Portland cement-stabilized earth blocks (OPC CEBs), which reduces their mechanical strength [44]. However, RC showed the same binding capacity of OPC, leading to similar mechanical strength if produced with the same porosity [44]. In addition, RC CEBs showed similar thermal and hygroscopic behavior to that of OPC CEBs [45,46]. Moreover, unlike unstabilized compressed earth blocks (UCEBs), RC CEBs maintained the integrity in water and exhibited high resistance to water erosion, making them suitable for unprotected outdoor applications [47,48].

However, although the good efficiency of RC as a CEB stabilizer has been demonstrated, its environmental performance has never been analyzed. The present research aims at quantifying the possible savings in energy consumption and carbon emissions obtained with RC-stabilized CEBs. The energy consumption and carbon emissions of UCEBs produced in the same context are also estimated for reference purposes, along with OPC CEBs.

## 2. Case Study

The CEBs were produced in Alcochete, Portugal, using natural soil extracted at the production site location. The soil (FA) for the CEB production, a silty-clayey sand [49], was enriched by adding clay powder (TV), a by-product of tile manufacturing from Cobert Company (Torres Vedras, Portugal). The blocks were stabilized with OPC, CEM I 42.5 R, and with Portland limestone cement (PLC), CEM II/B-L 32.5N [50]. The latter is a type of cement commonly used in low-strength applications, such as earth construction. The thermoactivated recycled concrete cement (RCC) was produced from concrete waste using an innovative magnetic separation method, as described in Carriço et al. [40]. The RCC was estimated to have 33 wt% of aggregate contamination, higher than usually achieved with this method (<25 wt% [40]). Due to this contamination, the stabilizer content (% by weight of FA + TV + CDW) in RCC mixtures was adjusted to match the same binder content of reference OPC CEBs. Water addition was set at 9% by weight of solids, except for CEBs with 100% RCC, which required 12%. In addition, earth was partially replaced by up to 40% CDW by volume, further enhancing the sustainability of CEBs (with the CDW content specified at the end of each mixture designation). The CDW was provided by the Portuguese recycling company Vimajas, Pêro Pinheiro. Nomenclature indicates the type of stabilizer (when used) followed by the CDW substitution rate; compositions referenced without “CDW” in nomenclature were produced with 25% CDW (Table 1).

The physical, mechanical, and durability characterization of the produced CEBs is reported elsewhere [47]. As discussed before, RCC CEBs presented higher porosity and lower mechanical strength than OPC CEBs due to their higher water demand. However, the rehydration and binding capacity of RCC were similar to those of OPC, reaching similar mechanical strength for the same porosity. Moreover, RCC significantly improved the water resistance of UCEBs, and RCC CEBs showed similar durability to OPC CEBs. A synthesis of the CEB composition, density (ρ_28,LC_), and unconfined compressive strength (f_c,un,lab_) under laboratory conditions at 28 days is presented in Table 1. For up to 50% replacement of OPC with RCC, strength loss was less than 20%.

## 3. Methodology and Data

### 3.1. Scope and Methodological Approach

**Figure 1:**
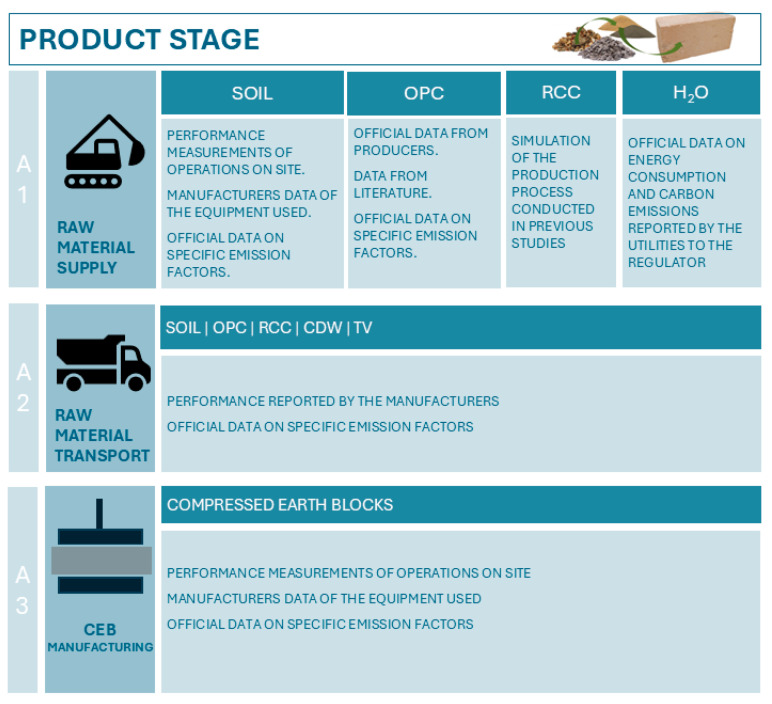
Scheme of the methodological approach.

In the present study, only the product stage, which included stages A1 to A3 according to the EN 15804 [51], was accounted for (Figure 1). The cradle-to-gate analysis focused solely on energy consumption and carbon emissions. The calculations were all performed by mass and converted to the functional unit of 1 CEB. The most common use of CEBs is in walls, which are measured in area for construction management purposes (e.g., bill of quantity estimates; billing and payments; schedule and cost control). However, the compaction degree of the blocks varies, implying differences in the raw materials and mechanical performance. Thus, the energy consumption and carbon emissions were also normalized per unit of compressive strength, to allow a fairer comparison.

The evaluation of the energy consumption and carbon emissions for the system was performed by simulation. The simulation model is hybrid, including external information and/or analogy approximations to account for certain processes (CDW, Portland cement, and recycled cement production), and direct simulation of other processes (soil extraction and processing and block production—preparation, mixture, and compaction).

### 3.2. Raw Materials (Supply and Transport)

Energy consumption and carbon emissions of each material supplied and transported (A1 and A2) for the CEB production were estimated. The soil accounted for its extraction by measuring the energy consumption on site. Official specific emission factors were used to estimate the corresponding carbon emissions. The recycled materials TV and CDW were used for CEB production as provided by the recycling companies with no additional processing; therefore, only the transportation was accounted for. The RCC required crushing and sieving, selecting, cleaning, magnetic separation, and thermal activation of the hydrated cement paste obtained. All the steps of RCC production are described in Carriço et al. [40], and the corresponding energy consumption and carbon emissions were estimated by Sousa et al. [42]. The production of OPC and PCL was calculated as the average of the most recently published environmental reports from the two main Portuguese cement producers and distributors [52,53,54,55,56]. Data for energy and carbon emissions of water from the public potable water distribution network were collected from suppliers on the yearly report of ERSAR [57], the Portuguese water and waste services regulation authority.

The transportation distances by road between the various locations were determined using Google Maps. The recent study from Mulholland et al. [58] was considered for modeling the corresponding fuel consumption and carbon emissions. Considering that in Portugal (and Europe) most road transportation vehicles are equipped with diesel engines, the energy content of diesel motor fuel of 38.290 kJ/L reported by the Bureau of Transportation Statistics [59] was adopted for calculating the energy consumption. It should be noted that it may slightly change according to the producer and other factors. The fuel conversion factor of 2.67 kgCO_2_/L for diesel was adopted [60,61] to convert the fuel consumption to carbon emissions. The vehicle considered for transportation was a VECTO 5-RD [58], with axle configuration of 4 × 2 and gross vehicle weight rating (GVWR) above 16 t with a load capacity of about 10 t, which is assumed to run about 90% under regional delivery. The average consumption of this truck group for the year 2020 was 31.7 L/100 km, with associated carbon emissions equal to 853.7 gCO_2_/km, according to Mulholland et al. [44].

### 3.3. Compressed Earth Block Manufacturing

The production phase entails the operations of mixing and pressing, and was modeled by the direct simulation of the operations taking place at the CEB production plant. The mechanical mixing requires an energy consumption of 3 kWh for the mixer, producing 21 CEBs in 4 min. This corresponds to an efficiency of 315 CEB/hour for the mixing device. The compression of the blocks was run with an Oskam V/F semiautomatic press (5–6 MPa) with an energy consumption of 7.5 kWh. According to the recorded data, the device can produce 1 CEB in 5 s, which corresponds to an efficiency of 720 CEB/hour.

The electrical energy consumed in CEB production was converted into carbon emissions using the 5-year moving average between 2018 and 2022 (198 gCO_2eq_/kWh), published by APA, the Portuguese Agency for the Environment [62]. The data show a significant decreasing trend of the carbon intensity of electricity production over the last decades, with a reduction from 519 gCO_2eq_/kWh in 1990 to 119 gCO_2eq_/kWh in 2023 [63].

**Figure 2:**
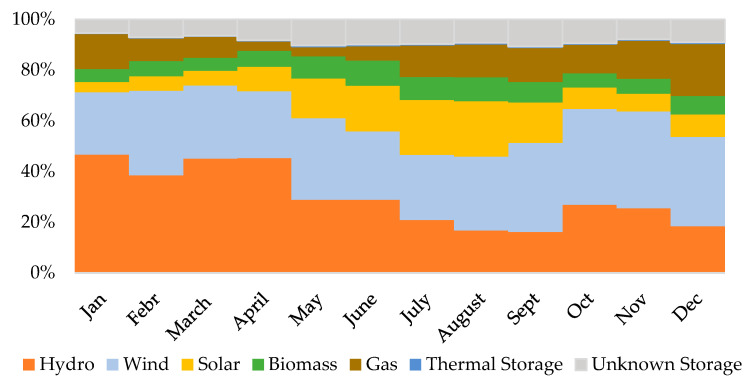
Monthly electricity origins for Portugal in 2024 (data from [51]).

In addition to the overall yearly carbon emissions reduction that has been taking place with the increase in the fraction of electricity produced from renewable sources and the shift to cleaner non-renewable sources, there is also a significant seasonal variation in the carbon intensity. The European Electricity Map [64] reports for Portugal 2024, from January to December 2024, a minimum and maximum carbon intensities of 67 gCO_2eq_/kWh (April) and 156 gCO_2eq_/kWh (December), respectively. Figure 2 displays the yearly electricity origins for Portugal in 2024, based on data from the national electrical grid operator [65], with the highest green energy production in April and the lowest in December, in agreement with data from the European Electricity Map. This implies that the concentration of the production of CEBs out of the winter months would offset their environmental benefits.

### 3.4. Scenarios

Four scenarios were considered in the study, representing the real case study (0), variations in it (1 and 2), and including a scenario more representative of traditional earth construction (3):

- Scenario 0—Real scenario. CEBs were produced in Montemor-o-Novo, where the manufacturing facility was settled. The soil FA was taken from Alcochete (excavation and transport) for the purpose of CEB production. The additional clay TV was retrieved from Torres Vedras, and CDW was brought from a recycling company in Pêro Pinheiro. OPC and PLC were produced at the SECIL cement plant in Outão, Setúbal. RCC was produced in Lisbon.
- Scenario 1—Same as the real scenario, but the soil was considered a by-product of excavation (for foundations), and RCC was also produced at the SECIL cement plant in Outão, Setúbal, and brought to Montemor-o-Novo.
- Scenario 2—CEBs were produced in Lisbon, and RCC was produced at the SECIL cement plant in Outão, Setúbal.
- Scenario 3—The production of CEBs occurred in the same place of soil extraction (Alcochete). The soil was retrieved as excavation waste, and earth correction (clay addition) was not necessary. OPC, PLC, and RCC were produced at any Portuguese cement plant.

**Figure 3:**
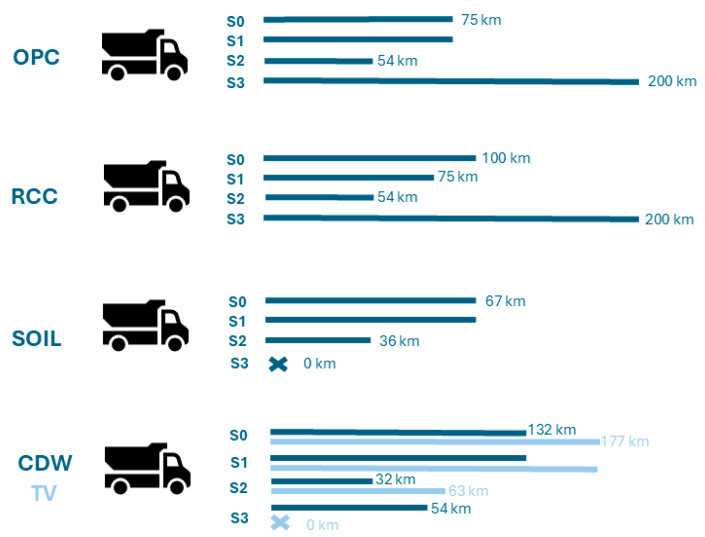
Transportation distances for each raw material in all scenarios.

These scenarios essentially introduce differences in terms of the transportation distances, as shown in Figure 3 and described in the following sections.

## 4. Results

### 4.1. Raw Materials

#### 4.1.1. Soil—FA

The soil for CEB production requires extraction; transportation; and, commonly, drying, crushing, and sieving. The soil used for earth construction traditionally has the advantages of being retrieved near the construction site (reducing the transportation costs) and, in the best-case scenario, being reused from foundation excavation [66]. Thus, one of the main advantages of earth construction material is the possibility of using local soil. Even when the composition includes some type of stabilizer, since the earth makes up most of the block mass, the overall transportation needs are still small. However, not any soil is adequate for CEB production, and if the facility is located at the construction site, the earth, or a portion of it, may need to be transported from other locations. Also, setting up the CEB production facility at the construction site requires space that might not be available in all cases. The scenarios considered different locations for the CEB production facility, to model the most likely configurations for excavation, transportation, and processing of the earth.

When the blocks are produced on the construction site, it is also avoided the transportation of the blocks (excluded from the present study). This is possible because the infrastructure needed for producing earth blocks is mobile, and some of the equipment (e.g., mixer, excavator) can be used in other construction works.

The modeling considered the equipment characteristics, from which the fuel consumption where measured/retrieved and emissions estimated, and the operation on site was measured to estimate the effective productivity—duration and amount of material for each soil processing phase was experimentally obtained. For the extraction, a Volvo EC290C was used, reporting a fuel consumption of 18.5 L/h and a productivity of 26 t/h. The energy consumption for soil extraction was estimated to be 27.04 MJ/t (or 0.00757 kWh/kg), consistent with the 0.00728 kWh/kg calculated by Christoforou et al. [67] or the ibidem referred value of 27 MJ per ton of sand indicated by Venta [68]. Transporting soil from the extraction site to the CEB production facility uses a category VECTO 5-RD truck [58]. The earth processing, namely the pulverizing and sieving steps, was run in the CEB production site, using equipment with electricity consumption of 1.1 and 4.1 kWh, respectively.

The energy consumption and carbon emissions to prepare the soil FA for CEB production were estimated for the functional unit established for the raw materials (1 t) and are displayed in Table 2.

#### 4.1.2. Clay Residue and Construction and Demolition Waste—TV and CDW

As mentioned, TV was obtained in the form of waste from a clay tile factory, so the extraction stage is not applicable. The same truck used for soil transportation (Section 4.1.1) was considered for transporting the tile residue, with the distance between the tile factory and the CEB production site ranging from 63 km in scenario S2 to 177 km in scenarios S0 and S1 (Table 3).

Similarly to TV, CDW is also a by-product and is assumed to be available without the need for further processing. Thus, this raw material only requires transportation from the recycling facility (which may be a construction site) to the CEB production unit. The present study assumed in all simulations that the material is sourced from the same recycling facility of Vimajas Company, located approximately 30 km northwest of Lisbon.

#### 4.1.3. Cement—OPC and PLC

The energy consumption from OPC and PLC was estimated from the official reports of Portuguese cement producers (Secil (Lisbon, Portugal) and Cimpor (Lisbon, Portugal)), resorting to the environmental declarations from each specific national production unit. From the six cement factories in the Portuguese mainland, only the data from one (Secil plant of Cibra-Pataias) were not included, since that plant only produced white cement over the last years. The data reports from 2023 were available for the other five production units except for the Cimpor plant of Souselas (the latest data from 2020). The data for each factory is presented in Table 4, along with the corresponding weighted average (based on the amount of clinker produced). The values are consistent with the environmental product declaration for Portuguese grey cement promoted by the Portuguese Technical Cement Association, which estimates thermal energy consumption at 3722.7 MJ/t of clinker and process emissions at 821.6 kg CO_2_/t of clinker [69]. The total electricity consumption (147.5 kWh/t cement) is split between the clinker (60%) and cement (40%) phases. Assuming that most of the electrical energy consumption in the cement stage is associated with clinker grinding, this corresponds to a specific consumption of 189.3 kWh/t clinker.

The energy consumption for gypsum and limestone filler was considered the same for the extraction and preparation of the raw material for cement production. In the Portuguese cement plants, an average of 12 MJ/t is spent with fossil fuels in the vehicles and equipment used for raw materials extraction [69]. According to the literature, the electricity consumption for the raw material processing corresponds to roughly 30% of the total needed for cement production [70,71,72]. The average carbon emissions estimation of 8.9 kgCO_2eq_/t for gypsum and limestone specific carbon emissions is consistent with literature (8 kgCO_2eq_/t reported by Bolte et al. [73]).

The clinker content was assumed to be 93% for OPC and 67% for PLC, with 3% gypsum incorporated for both, and 4% and 30% filler content, respectively.

For most scenarios, the transportation distances for OPC and PLC were calculated from the SECIL cement plant in Outão. Only for S3, representative of CEBs produced on site and stabilized with commercial cement, distances were estimated considering the number and location of the cement plants in Portugal. Five out of the six Portuguese cement plants are located in a region of approx. 200 km on the western coast between Setúbal and Souselas. The sixth is in the south (Algarve), roughly 250 km from the closest one on the western coast. With this cover, the maximum distance would be around 250 km from Sousela to the northeast corner of Portugal. However, considering that CEBs are more adequate for the dry and hot climate in the southern part of Portugal, the Outão (Secil) and Loulé (Cimpor) plants are the closest, and the maximum distance in a straight line to any location in this region of Portugal is, at most, 200 km. Table 5 displays the energy consumption and carbon emissions for the production and supply of both OPC and PLC, considering the boundary conditions of each scenario.

#### 4.1.4. Recycled Cement from Concrete Waste—RCC

The recycled cement is produced by extracting the hydrated cement paste from the aggregates of the concrete waste by magnetic separation. The method was presented in previous research efforts [40,42] and includes the following main stages: (i) crushing and milling of the concrete waste to obtain a material of particle sizes adequate for magnetic separation; (ii) dust removal from the graded material; (iii) magnetic separation, splitting the waste into hydrated cement paste particles and recycled aggregates with low cement paste content; and (iv) additional griding and thermal reactivation of the hydrated cement paste waste. The stage (ii) of dust removal was initially performed by washing the material, but the large energy consumption required for drying the material was found environmentally critical. Alternatives to this option were tested in Sousa et al. [43], and the air cleaning method was found to produce equivalent results with significantly lower energy consumption.

The energy consumption and carbon emissions associate with the production of RCC through the air cleaning method were already estimated in a previous studies [42,43] considering the analogy with clinker production and assuming that (a) the raw material (concrete waste) would be available from a construction and demolition waste treatment plant already pre-demolished, and therefore, only additional crushing and sieving would be required with an energy requirement of 1/3 of the energy used for crush and sieve the raw material for clinker production; (b) the cement paste waste (CPW) is separated from concrete waste at the CDW treatment plant and transported to the site where its thermal activation is planned, generally a cement plant (a conservative average distance of 100 km is assumed); and (c) the energy for the thermal treatment, both electrical and thermal, is estimated based on the temperature ratio 650 °C/1450 °C. The data in Table 6 was updated from previous research based on the latest data on clinker production in Portugal (Table 4) and fine-tuned, since no final milling was performed after the thermal reactivation. The particle size of the hydrated cement paste waste before thermal activation is below 250 μm, similar to the raw meal used in clinker production [74]. The thermal treatment further breaks down the particles by removing hydration water, and there is no agglomeration (no sintering reaction), resulting in a final material with a grading similar to Portland cement, below about 85 μm [75]. The total adjusted values presented in Table 6 were obtained by dividing the energy consumption and carbon emissions associated with RCC production by the fraction of reactivated cement paste in the mix, which was assumed to be 81% on average, based on values typically achieved using this method [42].

#### 4.1.5. The Water

Water supply management in Portugal follows a mixed model, with private and public companies operating the systems. In many regions, bulk (responsible for the abstraction, treatment, and supply) and retail (responsible for the distribution to the final consumer) water suppliers coexist, but water can also be provided by a single utility, which is the case in some regions. Regardless of the ownership of the water utility or the structure of the system, all the companies operating in the water sector in the country must report their performance yearly to the water, wastewater, and sewer service regulator [57], including the amount of water supplied and the energy consumption associated.

In the real case scenario (S0) and in scenario 1 (S1), the water is supplied by the Municipality of Montemor-o-Novo, which gets the bulk water from the Company Águas Públicas do Alentejo (Beja, Portugal). The two companies have a declared specific energy consumption of 0.370 (kWh)/m^3^ and 0.804 (kWh)/m^3^, respectively, resulting in a total of 1.174 (kWh)/m^3^. In scenario 2 (CEB production settled in Lisbon), water is supplied by the Empresa Portuguesa de Águas Livres (EPAL) (Lisbon, Portugal), and the specific energy consumption is 0.935 (kWh)/m^3^, given by the sum of 0.540 (kWh)/m^3^ and 0.395 (kWh)/m^3^ for the bulk and retail water supply, respectively. In the 3rd scenario, where the production of the CEBs is assumed in the same site of excavation and construction, the water is supplied by the Municipality of Alcochete with an energy consumption of 0.544 (kWh)/m^3^. All the energy consumption for water supply (Table 7) was calculated based on data from 2022, published by the water, wastewater, and sewer service regulator.

### 4.2. Manufacturing of Compressed Earth Blocks

Similarly to the approach used for modeling the earth supply, the production of CEBs combined equipment characteristics—such as fuel consumption and emissions—with on-site operational measurements to estimate effective productivity, including the duration and amount of CEBs produced. The energy consumption of the mixing process was estimated as 0.034 MJ/CEB, corresponding to 0.0019 kgCO_2eq_/CEB emissions. By adding 0.038 MJ/CEB and 0.0021 kgCO_2eq_/CEB associated with the use of the semiautomatic hydraulic press, the total energy consumption and carbon emissions for CEB production were estimated at 0.072 MJ/CEB and 0.004 kgCO_2eq_/CEB, respectively.

### 4.3. Product Stage

The estimated energy consumption and carbon emissions from CEB production are presented in Table 8. This was determined by combining the raw material supply and transportation with the production energy consumption and carbon emissions, weighted according to the various compositions.

Fernandes et al. [76] reported carbon emissions of 0.39 kg CO_2eq_ for producing one lime-stabilized CEB, which is slightly higher than the estimate for PLC CEBs in this study (0.32–0.37 kgCO_2_/CEB). The energy consumption was lower (1.90–2.58 MJ/CEB) compared to the 3.94 MJ/CEB reported by the same authors. Nevertheless, comparison is challenging due to the impact that modeling differences can have on the results, such as variations in the amount of stabilizer, transport distances, block dimensions, and other factors. The use of RCC improved the environmental performance of stabilized CEBs, particularly in terms of carbon emissions. This was expected, considering that a major advantage of RCC is the use of decarbonized raw material. In fact, the calcination of the calcium carbonate during the clinker production is responsible for roughly 2/3 of the total carbon emissions of the Portland cement, which is avoided in RCC produced from cement waste [43].

## 5. Discussion

### 5.1. General Considerations

As expected, the UCEBs are the best option in terms of environmental impact (energy consumption and carbon emissions were up to 93% and 99% lower than in OPC CEBs, respectively). However, UCEB applications are limited, essentially, to protected indoors. Regarding stabilized CEBs, for all the analyzed scenarios, the blocks with 100% RCC are associated with 6–8% lower energy consumption and 58–64% lower carbon emissions than those with 100% OPC. Smaller proportional reductions were attained for CEBs with 20% or 50% substitution of OPC with RCC.

The energy consumption associated with RCC stabilization leads to little environmental benefit in comparison to OPC if calculated as presented here. However, the separation process also produces high-quality recycled sand (HQRS), which accounts for roughly 18% of the concrete waste mass, alongside the production of RCC (about 6 wt%). Further HQRS can also be obtained by submitting the coarsest concrete waste fraction to magnetic separation (roughly 21% of the concrete waste mass). This fraction does not allow for obtaining pure cement paste particles, so it was not modeled herein.

The HQRS resulting from the magnetic separation stage was found to have less than 3 wt% of adhered paste [26], which is about six times lower than the cement paste typically adhered to untreated recycled concrete fine aggregates [77]. This corresponds to a water absorption of less than 2%, which is below the maximum water absorption of 3–5% recommended for good-quality recycled aggregates [78,79]. The remaining 50% consists of normal recycled sand (27 wt%) and normal recycled filler (23 wt%), which are CDW fractions that could be included in the RCC production. The material losses in the grinding and magnetic separation are roughly 5% in mass. Since HRQS makes up 18% of the material mass exiting the magnetic separator, and electricity consumption during thermal processing accounts for only 13% of the total, the electricity consumption allocated to the HQRS should correspond, at least, to roughly 75% of the total estimated for the RCC. This energy allocation to the two products would result in a reduction of 25% of the energy consumption and 20% of the carbon emissions attributed to the RCC.

Moreover, the reduction in carbon emissions of RCC CEBs compared to OPC and PLC CEBs can be further improved. The use of renewable energy sources to produce RCC is more feasible due to the lower temperature required to thermally activate the hydrated cement waste, potentially resulting in zero carbon emissions if renewable energy sources are used for electricity production. More importantly, except for the carbonation of the hydrated cement paste, the raw material of the RCC is virtually decarbonated. Portland cement production (OPC and PLC), on the other hand, generates significant carbon emissions from raw material decarbonation (525 kg CO_2_/t clinker from DAPHabitat [69]), which can only be reduced through carbon capture technologies or using the raw material in decarbonized form (e.g., chemical decarbonation of the carbonates prior to the thermal processing stage of the OPC production).

However, as discussed in Section 2, RCC CEBs tend to be produced with higher porosity and lower mechanical strength than OPC CEBs. Therefore, the environmental comparison must also consider the different technical performance between RCC and OPC CEBs, as analyzed in Section 5.3. Nevertheless, as mentioned, RCC CEBs showed water erosion resistance comparable to OPC CEBs, turning unstabilized CEBs from unsuitable for outdoor environments into water-resistant ones, regardless of the type of stabilizer (OPC or RCC) [47].

### 5.2. Impact of Product Stages and Scenarios

**Figure 4:**
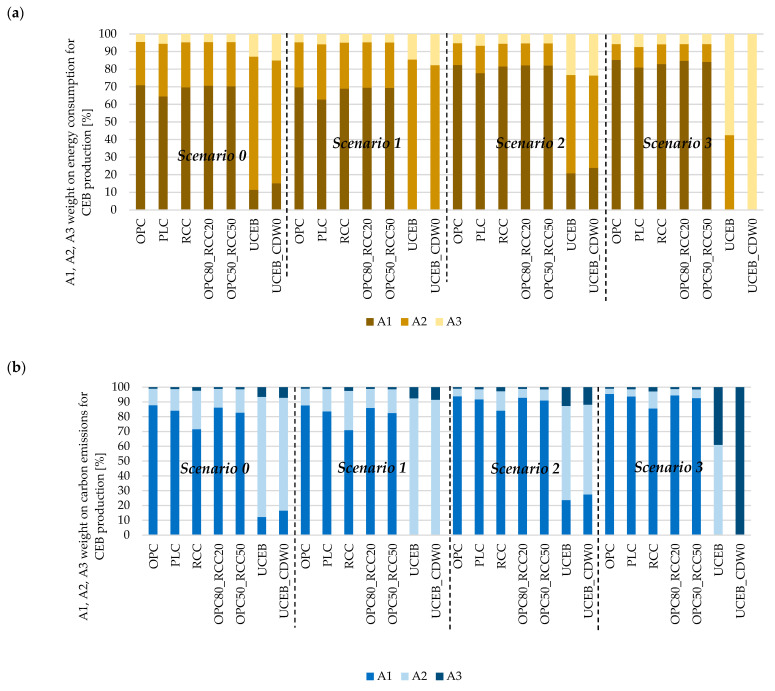
The impact of the product stages (raw materials supply—A1; transportation—A2; manufacturing—A3) on energy consumption (**a**) and carbon emissions (**b**) for CSEBs and UCEBs under each scenario.

The stabilization increased the energy consumption of raw materials supply (binder production, A1), with small differences between different scenarios (Figure 4a). The UCEBs, instead, were mainly affected by soil transport (A2) under scenarios S0, S1, and S2, and by manufacturing (A3) under scenario S3. Indeed, unstabilized CEBs have a high dependence on the transport distances of the modeled scenario, making their energy consumption calculations more likely to vary between studies.

Overall, similar results were observed in the analysis of the influence of the product stages (A1–A3) for each scenario in terms of carbon emissions (Figure 4b). The stabilization process increased the carbon emissions from raw material supply (A1), with a lower effect on CEBs stabilized with RCC. UCEBs had the highest impact on carbon emissions from transportation (A2), except in scenario 3, which modeled CEB production at the soil extraction site.

### 5.3. Normalized Environmental Impact

Stabilization significantly increased the mechanical strength of unstabilized CEBs by 3.8 and 2.6 times for OPC and RCC, respectively (f_c,un,LC_ of Table 1), demonstrating its efficiency. However, the RCC CEB compressive strength was 30% lower than that of OPC CEBs due to their higher water demand and consequent greater porosity. The use of additives to decrease the water demand and increase the CEB compactness is being explored to overcome this problem. In fact, as mentioned in Section 2, for the same total porosity, the compressive strength tends to be the same in OPC and RCC CEBs, because these stabilizers offer the same binding capacity [44]. The reduction in compressive strength in RCC CEBs was 14% compared to PLC CEBs. Finally, the partial replacement of OPC with 20% and 50% RCC led to 9% and 19% reduction in compressive strength (3.4 and 3 times higher than UCEB). This variation in performance between mixtures influenced the normalized environmental impact, represented by the ratio of energy consumption or carbon emissions per MPa of mechanical strength of CEBs (Figure 4).

As discussed in Section 5.1 (Table 8), the production of UCEBs had the lowest environmental impact, followed by PLC, RCC CEBs, and OPC CEBs. However, the ranking changes when the environmental impact is normalized to the compressive strength.

**Figure 5:**
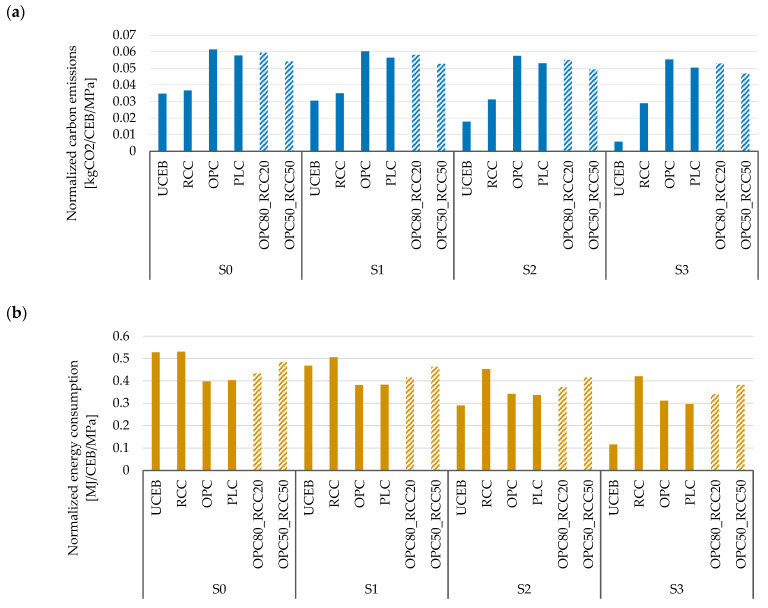
Normalized carbon emissions (**a**) and energy consumption (**b**) of CEBs for compressive strength (1 MPa).

In terms of carbon emissions, RCC CEBs still showed much better performance than OPC CEBs, despite their lower mechanical strength (Figure 5a).

On average, the normalized carbon emissions (NCE) were about 40–48% lower than in OPC CEBs, in all the analyzed scenarios. OPC and PLC had similar NCE across all the scenarios, with OPC slightly higher than PLC. As expected, the progressive substitution of OPC with RCC decreased the NCE. In scenarios S0 and S1, the NCE of RCC CEBs was only slightly higher than that of UCEBs. However, in scenarios S2 and S3, a decrease in NCE was observed for UCEBs, especially in scenario S3, where neither excavation nor transportation of the soil was accounted for. Nevertheless, UCEBs had the best performance under all the scenarios.

Regarding the normalized energy consumption (NEC), RCC performed worse than OPC CEBs (Figure 5b). On average, the NEC of OPC CEBs and PLC CEBs was 25–26% lower than that of RCC CEBs. In fact, the NEC of all CEBs stabilized with different binders fell within the same range of values in each scenario, with a standard deviation of 0.04–0.05 MJ/CEB/MPa. The NEC increased with the partial substitution of OPC by RC, leading to values intermediate to those obtained with OPC or RCC alone. As discussed in Section 5.1, NEC would be lower in RCC CEBs if HQRS production were also considered. UCEB performance, instead, is greatly affected by the analyzed scenario due to the high impact of soil excavation and transportation. UCEBs had the lowest NEC when produced closer to the soil extraction site (S2 and S3), reflecting the inherent philosophy of traditional earth construction.

From this study, it is concluded that RCC can be a viable alternative stabilizer to OPC, reducing the carbon footprint of CEBs, without significantly affecting its stabilization efficiency and durability performance. Moreover, with the production of RCC featuring lower aggregate contamination (as already achieved in [40]) and lower water demand, RCC CEBs are expected to be more environmentally friendly, offering higher compressive strength and lower stabilizer content. Considering that stabilization is required for most outdoor applications, RCC CEBs potentially offer the best compromise between technical performance and eco-efficiency.

### 5.4. Influence of CDW Incorporation

**Figure 6:**
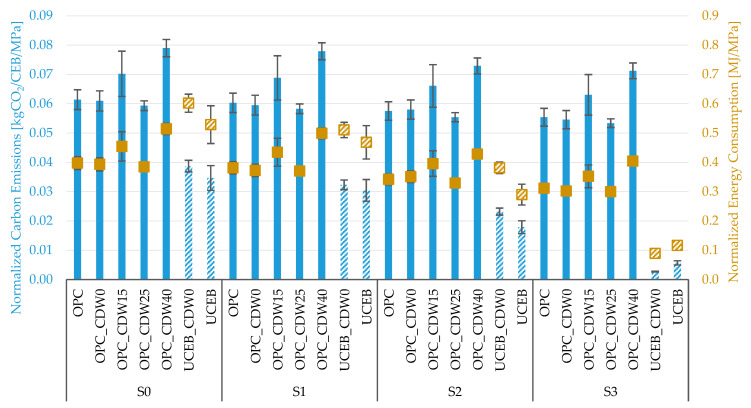
Normalized (f_c,un,LC_ average and standard deviation) carbon emissions and energy consumption for compressive strength of CEBs with different rates of CDW incorporation.

The effect of CDW incorporation on NCE and NEC is presented in Figure 6. The highest compressive strength in OPC CEBs was achieved with partial replacement of earth by 0% and 25% CDW (Table 1). This is likely due to the higher compactness attained in CEBs with 25% CDW [33]. The same occurred in UCEBs. The statistical significance of results within the same scenario was confirmed through ANOVA analysis, with *p*-values ranging between E^−06^–E^−14^ for NEC and E^−19^–E^−12^ for NCE.

In general, the energy consumption and carbon emissions slightly increased with CDW content, both in OPC CEBs and UCEBs (Table 8). The exception occurred in scenario S2 because the transport distance was about the same for CDW and earth (Figure 3). In all the other scenarios, the transport distance for CDW was 65 km (S0, S1) and 54 km (S3) higher than for FA. The CDW from the company located 30 km northwest of Lisbon could be replaced with CDW from a company closer to the production site, thereby reducing the transport-related environmental impact. The NEC and NCE of stabilized CEBs were negatively affected by the addition of CDW at 15 and 40% due to the corresponding reduction in compressive strength (Figure 5). UCEBs with 25% CDW presented the lowest values of NEC and NCE in scenarios S0, S1, and S2. Only in Scenario S3, where the soil was retrieved from foundation excavation and CEB production was in the same excavation/construction site, the gain in compressive strength was not enough to turn into a more environmentally beneficial solution—the addition of CDW to unstabilized CEBs.

## 6. Conclusions

The present study analyzed the energy consumption and carbon emissions of CEBs stabilized with recycled cement sourced from concrete waste (RCC) and compared them with CEBs stabilized with ordinary Portland cement (OPC) or Portland limestone cement (PLC), and with unstabilized CEBs. CEBs were produced with up to 40% earth replacement with CDW. Due to aggregate contamination, RCC CEBs required 50% more stabilizer content than OPC CEBs. Additionally, because of its higher water demand, RCC CEBs exhibited higher porosity and lower mechanical strength. These aspects are the focus of ongoing research aimed at further improving the environmental performance of RCC CEBs.

A cradle-to-gate analysis was conducted, considering the three (A1–A3) modules of the product stage. This was based on a hybrid model that relied on external information to estimate the energy consumption and carbon emissions of certain raw materials (OPC, PLC, and RCC) and directly simulated the supply and transportation of FA, TV, and CDW. Finally, the manufacturing of compressed earth blocks (CEBs) was quantified through direct simulation.

The effect of each production stage on the final energy consumption and carbon emissions was evaluated, revealing that UCEBs are more influenced by the selected scenario than CSEB, primarily due to the significant weight of the transport stage (A2) in UCEB production. The addition of up to 25% CDW had little effect on the environmental performance of CSEB.

The lowest energy consumption and carbon emissions were obtained for unstabilized CEBs (UCEBs), ranging 0.2–1.1 MJ/CEB and 0.01–0.07 kgCO_2_/CEB, respectively. However, UCEBs are limited to protected indoor solutions. As expected, the stabilization had the overall effect of increasing both energy consumption and carbon emissions. Compared to UCEBs, energy consumption and carbon emissions increased by 2–9 times and 5–35 times in OPC CEBs, respectively. However, substituting OPC with RCC resulted in a 6–8% reduction in energy consumption and a 58–64% reduction in carbon emissions. The environmental benefit of RCC CEBs can be significantly enhanced if the simultaneous production of high-quality recycled sand is also considered, along with the adoption of renewable energy resources.

Due to the lower mechanical strength of RCC CEBs, the normalized energy consumption was up to 35% higher in RCC CEBs compared to OPC CEBs, but carbon emissions remained 40–48% lower. As the binding capacity of RCC and OPC is similar, these ratios can be improved for RCC CEBs produced with the same porosity as OPC CEBs. It was concluded that RCC can be a viable alternative stabilizer to OPC, effectively reducing the carbon footprint of CEBs.

It should be noted that the normalization adopted only considered the difference in mechanical resistance. Another significant aspect that is commonly improved with stabilization is durability. Herein, it was assumed that CEBs would only be used in indoor applications, considering the specific case of equal durability between unstabilized and stabilized blocks. However, exposure to outdoor conditions could happen in different designs, and considering the long-life cycle of buildings, this aspect may have a significant role in the overall assessment of the environmental performance of CEBs. Further research is needed to fully understand the durability differences and their impact on environmental performance.

## Figures and Tables

**Table 1:** Synthesis of the CEB compositions, bulk densities, and unconfined compressive strength.

Designation	FA (kg/m^3^)	TV (kg/m^3^)	OPC ^(a)^ (kg/m^3^)	RCC (kg/m^3^)	CDW (kg/m^3^)	H_2_O_Tot_ (kg/m^3^)	ρ_f_ (kg/m^3^)	ρ_28,LC_ (kg/m^3^)	f_c,un,LC_ (MPa)
OPC	1244	153	144	-	400	211	2155	2064	7.9
OPC_CDW0	1612	196	144	-	0	200	2169	2103	7.9
OPC_CDW15	1362	200	144	-	239	207	2153	2076	6.9
OPC_CDW25	1195	202	144	-	400	211	2159	2084	8.2
OPC_CDW40	932	207	144	-	654	215	2179	2110	6.2
PLC	1244	153	144 *	-	400	211	2157	2070	6.4
RCC	1139	138	-	197	367	256	2114	2018	5.5
OPC80_RCC20	1235	151	114	43	398	211	2122	2038	7.2
OPC50_RCC50	1222	149	71	105	394	211	2118	2047	6.4
UCEB	1253	272	-	-	438	216	2188	2001	2.1
UCEB_CDW0	1623	351	-	-	-	205	2195	2075	1.8

Notation: FA—soil; TV—lean clay; CDW—construction and demolition waste; OPC—ordinary Portland cement; RCC—recycled concrete cement; ρ_f_—fresh density; ρ_28,LC_—bulk density at laboratory conditions; f_c,un,LC_—unconfined compressive strength at laboratory conditions. ^(a)^ CEM I 42.5R except for * CEM II/B-L 32.5N.

**Table 2:** Energy consumption and carbon emissions for the supply of the raw material: soil FA.

The Soil FA				
Energy Consumption [MJ/t]	S0	S1	S2	S3
Extraction	27.2	-	27.2	-
Transportation	80.1	80.1	43.7	-
Processing	14.5	14.5	14.5	14.5
Total Energy [MJ/t]	121.8	94.6	85.4	14.5
**Carbon Emissions [kgCO_2_/t]**				
Extraction	1.90	-	1.90	-
Transportation	5.64	5.64	3.07	-
Processing	0.14	0.14	0.14	0.14
Total Carbon Emissions [kgCO_2_/t]	7.68	5.78	5.11	0.14

**Table 3:** Energy consumption and carbon emissions for the supply of raw materials: TV and CDW.

	TV				CDW			
Energy Consumption [MJ/t]	S0	S1	S2	S3	S0	S1	S2	S3
Transportation	209.4	209.4	74.1	-	156.9	156.9	37.7	63.9
Total Energy [MJ/t]	209.4	209.4	74.1	-	156.9	156.9	37.7	63.9
**Carbon Emissions [kgCO_2_/t]**								
Transportation	14.73	14.73	5.21	-	11.0	11.0	2.7	4.5
Total Carbon Emissions [kg CO_2_/t]	14.73	14.73	5.21	-	11.0	11.0	2.7	4.5

**Table 4:** Energy consumption and carbon emissions for clinker and calcium carbonate filler productions [38,39,40,41,42].

Clinker Production								
	Secil			Cimpor				PT Av ^(1)^
Cement Plant	A	B	Av	C	D	E	Av	
Energy Consumption [MJ/t clinker]								
Thermal Energy	3790.0	3892.4	3825.7	3621.3	3778.4	3528.6	3600.7	3689.7
Electrical Energy	523.3	611.8	554.2	501.0	550.9	442.9	482.3	510.7
Total	4313.3	4504.1	4379.9	4122.3	4329.3	3971.5	4083.0	4200.4
Carbon Emissions [kgCO_2_/t clinker]								
Thermal Energy	*799.5*	786.4	794.9	817.0	810.0	819.0	817.0	808.3
Electrical Energy	28.8	33.7	30.5	27.6	30.3	24.4	26.6	28.1
Total *	828.3	820.1	825.4	844.6	840.3	843.4	843.6	836.4
**Gypsum and Calcium Carbonate Filler Production**								
Energy [MJ/t filler]	162.0	187.4	170.9	155.6	170.0	139.0	150.3	158.4
Carbon Emissions * [kg CO_2_/t filler]	9.1	10.5	9.6	8.7	9.5	7.8	8.5	8.9

Notation: Av—average value; * values calculated using the conversion factor for Portugal 0.198 kgCO_2eq_/kWh [48]; ^(1)^ weighted average calculated considering the clinker production and the energy declaration from the cement plant (environmental reports 2023).

**Table 5:** Energy consumption and carbon emissions for the supply of the raw materials: OPC and PLC.

	OPC				PLC			
Energy Consumption [MJ/t]	S0	S1	S2	S3	S0	S1	S2	S3
Production	3917.5				2866.6			
Transportation	89.9	89.9	64.3	236.6	89.9	89.9	64.3	236.6
Total Energy [MJ/t]	4007.3	4007.3	3981.8	4154.1	2956.4	2956.4	2930.8	3103.2
**Carbon Emissions [kgCO_2_/t]**								
Production	778.5				563.5			
Transportation	6.3	6.3	4.5	16.6	6.3	6.3	4.5	16.6
Total Carbon Emissions [kg CO_2_/t]	784.8	784.8	783.0	795.1	569.8	569.8	568.0	580.1

**Table 6:** Energy consumption and carbon emissions for the supply of the raw materials: RCC.

RCC Production				
Energy Consumption [MJ/t]	S0	S1	S2	S3
Thermal energy	1302.5			
Electrical energy	686.9			
Transportation of separated CPW	121.4			
*Adjusted Total for Production* *	2615.0			
Transportation of RCC	118.8	89.5	64.3	236.6
*Total Energy [MJ/t]*	*2733.8*	*2704.6*	*2679.3*	*2851.6*
**Carbon Emissions [kgCO_2_/t]**				
Thermal energy	103.8			
Electrical energy	37.8			
Transportation of separated CPW	8.5			
*Adjusted Tot for production* *	185.8			
Transportation of RCC	8.4	6.3	4.5	16.6
*Total Carbon Emissions [kg CO_2_/t]*	194.2	192.2	190.4	202.5

Notation: * total value adjusted by the degree of cement purity content (81% on average).

**Table 7:** Energy consumption and carbon emissions for the supply of the raw material: water.

Water			
	S0, S1	S2	S3
Total Energy [MJ/t]	4.23	3.37	1.96
Total Carbon Emissions [kg CO_2_/t]	0.23	0.19	0.11

**Table 8:** Energy consumption and carbon emissions for CEB production.

Designation	Total Energy [MJ/CEB]				Total CO_2_ Emissions [kgCO_2_/CEB]			
	S0	S1	S2	S3	S0	S1	S2	S3
OPC	3.14	3.01	2.70	2.46	0.485	0.476	0.454	0.438
OPC_CDW0	3.11	2.94	2.77	2.39	0.481	0.470	0.458	0.431
OPC_CDW15	3.14	3.00	2.73	2.43	0.484	0.475	0.456	0.435
OPC_CDW25	3.15	3.03	2.70	2.46	0.486	0.478	0.454	0.438
OPC_CDW40	3.19	3.09	2.65	2.51	0.490	0.483	0.452	0.442
PLC	2.58	2.45	2.15	1.90	0.370	0.361	0.339	0.322
RCC	2.92	2.78	2.49	2.31	0.202	0.192	0.172	0.159
OPC80_RCC20	3.12	2.99	2.68	2.45	0.428	0.419	0.397	0.381
OPC50_RCC50	3.10	2.97	2.66	2.45	0.347	0.337	0.315	0.301
UCEB	1.11	0.98	0.61	0.24	0.073	0.064	0.038	0.012
UCEB_CDW0	1.08	0.92	0.69	0.16	0.070	0.058	0.042	0.005

## Data Availability

The original contributions presented in this study are included in the article. Further inquiries can be directed to the corresponding author.

## Abbreviations

The following abbreviations are used in this manuscript:

CDW	Construction and demolition waste
CEB	Compressed earth block
NCE	Normalized carbon emissions
NEC	Normalized energy consumption
OPC	Ordinary Portland cement
RC	Recycled cement
RCC	Recycled cement from concrete waste
UCEB	Unstabilized compressed earth block
